# From DNA Repair to Redox Signaling: The Multifaceted Role of APEX1 (Apurinic/Apyrimidinic Endonuclease 1) in Cardiovascular Health and Disease

**DOI:** 10.3390/ijms26073034

**Published:** 2025-03-26

**Authors:** Huan-Huan Yuan, Hao Yin, Mara Marincas, Ling-Li Xie, Lan-Lan Bu, Min-Hua Guo, Xi-Long Zheng

**Affiliations:** 1 Key Laboratory of Hunan Province for Integrated Traditional Chinese and Western Medicine on Prevention and Treatment of Cardio-Cerebral Diseases, College of Integrated Traditional Chinese and Western Medicine, Hunan University of Chinese Medicine, Changsha 410208, China; 2 Department of Biochemistry & Molecular Biology, Libin Cardiovascular Institute, Cumming School of Medicine, University of Calgary, Calgary, AB T2N 4Z6, Canada; 3 Robarts Research Institute, Schulich School of Medicine and Dentistry, Western University, 1151 Richmond St. N., London, ON N6A 5B7, Canada

**Keywords:** APEX1, redox activity, endonuclease activity, DNA repair, atherosclerosis, cardiovascular diseases

## Abstract

Apurinic/apyrimidinic endonuclease 1 (APEX1) serves as a potent regulatory factor in innate immunity, exhibiting both redox and endonuclease activities. Its redox function enables the regulation of transcription factors such as NF-κB or STAT3, whereas its endonuclease activity recognizes apurinic/apyrimidinic (AP) sites in damaged DNA lesions during base excision repair (BER) and double-stranded DNA repair, thereby I confirm.anti-inflammatory, antioxidative stress and antiapoptotic effects. APEX1 is expressed in a variety of cell types that constitute the cardiovascular system, including cardiomyocytes, endothelial cells, smooth muscle cells, and immune cells. Emerging genetic and experimental evidence points towards the functional roles of APEX1 in the pathophysiology of cardiovascular diseases, including neointimal formation and atherosclerosis. This review aims to present comprehensive coverage of the up-to-date literature concerning the molecular and cellular functions of APEX1, with a particular focus on how APEX1 contributes to the (dys)functions of different cell types during the pathogenesis of cardiovascular diseases. Furthermore, we underscore the potential of APEX1 as a therapeutic target for the treatment of cardiovascular diseases.

## 1. Discovery and Structure of APEX1

Apurinic/apyrimidinic endonuclease 1 (APEX1), also known as APE or redox factor 1 (Ref-1), is an endonuclease found in mammalian cells, recognized for its ability to detect and cleave baseless sites generated either spontaneously or by damage-specific DNA glycosylases [1].

Early 1970s–1980s (Initial Discovery)**.** During this era, scientists identified spontaneous depurination and depyrimidination events that threatened genomic stability. The concept of apurinic/apyrimidinic (AP) sites as intermediates in DNA repair began to emerge. Research teams discovered an unidentified endonuclease capable of cleaving AP sites in both bacterial and eukaryotic cells. By 1981, Demple et al. and Linn et al. showed that a mammalian AP endonuclease could efficiently process such sites [2,3,4,5].

**Figure 1:**
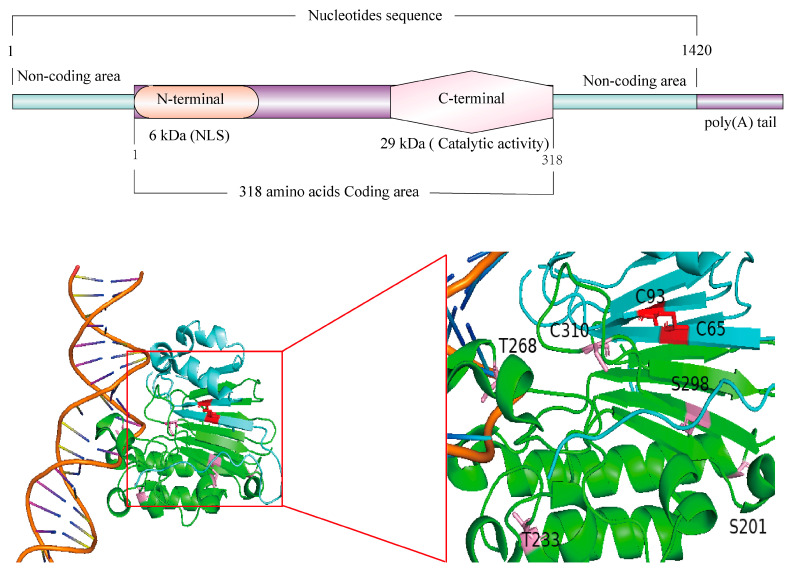
Domain Structure of the Human APEX1 Protein. The above is the structural model diagram of APEX1. The diagram below is the 3D structural model, with the 3D crystal structure obtained from the PDB database. The blue region represents the redox domain of APEX1 (The red region, C63 and C93, are the active sites of the redox domain), while the green region represents the endonuclease domain (The pink regions are active site of the endonuclease domain).

Late 1980s–1990s (Molecular Characterization). Significant progress led to the purification and characterization of this enzyme, now termed APEX1 [6]. In 1986, Spiro et al. first cloned the human APEX1 gene [7], and Seki et al. subsequently identified a cDNA from a human bone marrow library encoding a nuclear protein homologous to mouse APEX1 [8]. The human APEX1 cDNA spans 1420 nucleotides, including a 205-nt 5′-UTR, a 954-nt coding region (318 amino acids), and a 261-nt 3′-UTR (Figure 1) [8]. Early biochemical work indicated that the mature enzyme lacks an N-terminal methionine and often cleaves into 31, 33, and 35 kDa fragments during isolation. Two major domains were defined: a 6 kDa N-terminal region containing the nuclear localization signal (NLS) and a 29 kDa C-terminal catalytic domain [8,9].

Early 2000s (Structural Analyses). Crystallographic and mutagenesis studies illuminated the active-site geometry, substrate specificity, and catalytic residues essential for AP site cleavage [10,11]. Zhao et al. localized the human APEX1 gene to chromosome 14q12 [12]. X-ray–induced and site-directed mutagenesis indicated that APEX1 has a spherical C-terminal endonuclease domain and an N-terminal domain essential for its redox activity [13,14]. The four-layer α/β sandwich core structure belongs to the phosphoesterase superfamily, which directly binds the DNA backbone to survey and incise AP sites [15,16,17].

## 2. Functions of APEX1

### 2.1. N-Terminal Domain DNA/RNA/Protein Binding and Redox Activity

The N-terminal domain of APEX1 incorporates an NLS and demonstrates redox activity [18]. This unstructured region, encompassing residues 1–127, primarily serves a transcriptional co-activation role [18]. Mammalian APEX1 redox activity has been localized to the initial 127 residues, potentially involving Cys65 and Cys93. The removal of residue 61 from the N-terminus significantly diminishes the DNA-binding capacity of APEX1 [18,19,20,21]. Moreover, a NLS in the first 20 amino acids of APEX1 is indispensable for interaction with importin α1 and importin α2 [22], the major proteins for active nuclear transport. Fantini et al. identified five lysine residues (K24, K25, K27, K31, and K32) in the APEX1 N-terminus that interact with RNA and nucleophosmin1 (NPM1), a protein chaperone that controls nucleus-to-cytoplasm transport [23]. These lysine residues can be acetylated to regulate protein or RNA binding of APEX1 in cultured cells [23], which play essential roles in coordinating and optimizing BER activity and RNA metabolism [23]. Intriguingly, the dual RNA and protein binding capacity constitutes an important role of APEX1 in microRNA metabolism under genotoxic stress via physical interaction with the DROSHA-processing complex [24]. Moreover, X-ray crystal structure analysis demonstrates that the amyotrophic lateral sclerosis-associated polymorphism L104R in the N-terminus impacts APEX1 DNA recognition, reducing its AP site incision capacity [25]. APEX1 predominantly recognizes AP lesion sites in double-stranded DNA, but it also exhibits activity at AP sites in other environments, such as single-stranded DNA or G-quadruplex DNA [26]. G-quadruplexes can occur in many genomic regions, such as promoters and telomeres, both of which are sites of DNA oxidation where BER is active [27]. AP-containing four-track telomere G4 folds are substrates for APEX1 to cleave the baseless site [28]. The magnesium metal ion is an important factor in promoting the configuration change of the APEX1 N-terminus, which affects its DNA recognition activity to regulate its base repair and transcriptional capacities [29,30]. Recently, Fleming et al. demonstrated that G4 folds bearing AP sites are poorly cleaved by APEX1, but they are bound by the protein with a high affinity represented by low dissociation constants (KD) [31]. The KD values were negatively correlated with Mg2+ concentration, demonstrating that the divalent metal yielded stronger binding affinity [31,32]. In line with this, Howpay Manage et al. reported that without Mg(OAc)2, the KD values increased, suggesting Mg^2+^ facilitates APEX1 binding to G4 folds found in the telomere sequence and the endonuclease activity was also diminished [26]. Importantly, early studies have indicated a recognition function of the N-terminal tail of APEX1 in the enzyme’s proficiency in detecting conformational distortions in the DNA helical structure from diverse substrates, including AP sites, DNA strand break ends, and base mismatches [13,33,34]. It is important to note that, in addition to DNA lesions, APEX1 is also active, conceivably with different binding and/or catalytic properties, at AP sites in other environments, such as single-stranded DNA or G4-stranded DNA. Such a significant diversity of APEX1 substrates indicates that the enzyme can detect conformational distortions in the DNA helical structure [13,35], albeit the molecular and structural actions for how APEX1 processes non-DNA lesion substrates remain elusive.

**Figure 2:**
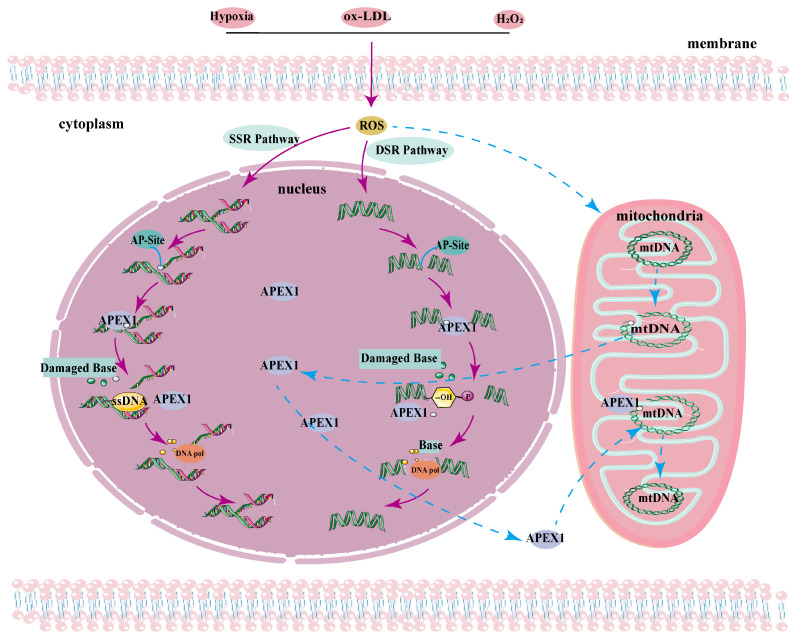
APEX1 repairs DNA damage mechanisms. Oxidative stress, hypoxia, and other factors lead to the production of a large amount of reactive oxygen species (ROS) within cells, resulting in damage to nuclear DNA and mitochondrial DNA. DNA damage in the nucleus exposes AP sites, which are recognized and bound by APEX1. APEX1 cleaves the AP site, and with the involvement of DNA glycosylase, the damaged base is excised, generating a -OH group at the 3′ end of the damage site. DNA repair is then carried out with the participation of DNA polymerase and DNA ligase. In the case of mitochondrial DNA damage, APEX1 is translocated to the mitochondria through a series of signal transduction processes to facilitate DNA repairs. (DSR, double-strand break repair pathway; mtDNA, mitochondrial DNA; SSR, single strand repair. The blue arrow represents APEX1’s involvement in the mitochondrial DNA repair pathway, while the purple arrow represents APEX1’s involvement in the nuclear DNA repair pathway.

One of the best-recognized functions of APEX1 is its role in BER (Figure 2). As we discuss in the following section, the C-terminal endonuclease of APEX1 constitutes the enzymatic component of its DNA repair activity, but its N-terminus plays a critical role in the interaction and coordination with multiple enzyme components in the BER cascade. The BER complex is initiated by the scaffold protein, X-ray repair cross-complementing 1 (XRCC1), which brings other enzymes, including APEX1, to the site of damage [36,37,38]. Of note, the first 35 residues of the APEX1 N-terminus are essential for the physical interaction with and targeted recruitment by XRCC1 [36]. APEX1 then stimulates the activity of DNA glycosylase such as 8-Oxoguanine Glycosylase 1 (OGG1) and facilitates the release of the cleavage products from DNA glycosylase, while DNA polymerase β (Pol β) and XRCC1 promote APEX1 turnover, with APEX1’s N-terminal tail playing an integral role [36,39,40]. The release of cleaved DNA products is the rate-limiting step for APEX1 activity, and a truncated APEX1 missing the N-terminal 33 residues exhibits reduced affinity for cleaved DNA but is marginally stronger than wild-type APEX1. This suggests that APEX1’s N-terminus is responsible for retaining APEX1 on the reaction product [23,41,42]. By binding to the N-terminal tail of APEX1, XRCC1 appears to aid in detachment of APEX1 from DNA, thus facilitating turnover [36].

APEX1’s N-terminal tail can also mediate redox activation of different transcription factors [43], including signal transducer and activator of transcription 3 (STAT3), activator protein 1 (AP-1) [44], hypoxia-inducible factor (HIF)-1α [45], and nuclear factor (NF)-κB [46]. Through interplay with these transcription factors, APEX1 executes its functions in various cellular processes, including cell survival, growth, motility, and inflammation [46]. For example, although APEX1 does not directly bind to the AP-1 sequence motifs recognized by Fos and Jun, it co-purifies with AP-1 from HeLa nuclear extracts using sequential heparin-Sepharose column purification and blue Sepharose chromatography [46]. APEX1 can activate the DNA binding activity of AP-1 through its conserved cysteine residues while maintaining AP-1 DNA binding specificity [6]. The APEX1-AP-1 interplay has significant outcomes in tumor cell behaviors and the immune system [47]. For instance, E3330, a selective inhibitor for the redox function of APEX1, suppresses AP-1 DNA binding and subsequent expression of cytokines in RAW264.7 macrophage-like cells challenged with lipopolysaccharide (LPS) [48]. Another good example is the regulation of STAT3 by APEX1. The DNA binding ability of STAT3 is redox-sensitive. Exogenous reduced APEX1 protein can increase STAT3-DNA binding in electrophoretic mobility shift assays with pancreatic tumor cell nuclear extracts [49]. E3330 can abrogate the DNA binding and transcriptional activity of STAT3 in pancreatic tumors [49]. Blocking APEX1 redox activity synergizes with STAT3 inhibitor to enhance tumor cell apoptosis [49]. Wang et al. also discovered similar functions in endothelial cells. Overexpression of APEX1 in endothelial cells can inhibit hypoxia and TNFα-induced apoptosis through NF-κB-independent or dependent pathways. Additionally, the loss of the N-terminal oxidoreductase domain of APEX1, which is primarily known for its role as an endonuclease in the BER pathway, increases the sensitivity of endothelial cells to TNFα and hypoxia-induced apoptosis, indicating that endogenous APEX1 plays a critical protective role in the vascular system [50]. All these transcription factor-related redox functions will be further discussed in the following sections on cardiovascular diseases and cell biology.

### 2.2. C-Terminal Domain Endonuclease Activity

Human APEX1 is a multi-functional DNA repair enzyme that shares a 26% sequence homology with Escherichia coli endonuclease III (Exo III). Analogous to Exo III, APEX1 functions as an endonuclease localized to its C-terminus, which hydrolyzes the 5′ AP site on the DNA backbone during BER [8,51,52,53,54]. Based on this enzymatic activity, APEX1 can repair DNA damage induced by various genotoxic stress, such as X-rays and hydrogen peroxide H_2_O_2_ [53]. Reactive oxygen species (ROS), which are by-products of respiration, cause DNA damage that includes oxidative bases, abasic sites, and oxidized AP sites. These types of damage are repaired through the BER, a critical cellular mechanism for maintaining DNA stability and integrity, both in the nucleus and mitochondria. Notably, the mitochondrial genome is especially susceptible to ROS-induced damage. Szczesny et al. observed the repair of damaged bases and AP sites involving single nucleotide binding using both mitochondrial and nuclear extracts [55]. The BER pathway mends AP site lesions by excising and replacing them with standard nucleotides via template-directed DNA synthesis. A group of specialized scaffold proteins and enzymes comprise the BER pathway. Utilizing a sensitive and specific photoaffinity labeling probe with a nicked DNA structure and a reduced abasic sugar site, Lavrik et al. identified a complex consisting of poly (ADP-ribose) polymerase 1 (PARP-1), three closely related BER factors—Flap Structure-Specific Endonuclease 1 (FEN1), POLB (DNA polymerase β), and APEX1 [56]. Although APEX1 was not co-immunoprecipitated with PARP-1, FEN, or POLB, this study provides strong evidence to support the direct role of APEX1 in physical interaction with damaged DNA as a part of multi-component BER machinery.

Masuda et al. discovered that because the enzymatic product of AP endonuclease acts as a competitive inhibitor of APEX1 in BER, Pol β is responsible for capturing the product of APEX1 activity to sustain its actions. In addition, the site-directed mutation of D283A and D308A in the APEX1 C-terminus revealed a segregation of DNA binding and DNA incision abilities [57]. For the detailed molecular actions of APEX1 in BER and other nucleic acid cleavage processes, we recommend a recent comprehensive review by Whitaker and Freudenthal [35]. Importantly, oxidized DNA lesions, especially in the form of 8-oxoguanine, can delay the progression of RNA polymerase, causing the transcriptional arrest, which has been recognized as an epigenetic signal derived from oxidative stress [58]. The coordinated repair of oxidized DNA damage by OGG1 and APEX1 is an integral component of redox regulation of epigenetics, which we will discuss in the following sections on cardiovascular pathophysiology. Here, we briefly present an example from a seminal study by Fleming et al. [59]. In cultured mouse embryonic fibroblasts, ROS-induced DNA oxidation generates 8-oxoguanine, which leads to a threefold increase in gene transcription of VEGFA and NTHL1. The transcriptional activation involves 8-oxoguanine-dependent formation of G4 folds in the promoter DNA strand, which initiates BER by OGG1 and produces an AP site that allows the binding of APEX1. The persistent presence of APEX1 in the gene promoter results in transcriptional activation, albeit the recruited transcriptional machine remains to be further investigated. Intriguingly, it is the DNA binding, instead of DNA cleavage, ability of APEX1 that is important to the transcriptional activation, as APEX1 inhibitor III, which specifically prevents APEX1 cleavage of the DNA backbone, augments APEX1-dependent transcriptional activation by >10-fold [59].

### 2.3. 3′-Exonuclease Activity

APEX1 also possesses a 3′–exonuclease activity, which preferentially targets mismatched nucleotides at the 3′ ends of nicked DNA, enhancing the fidelity of DNA repair [60]. This activity is crucial for removing potentially mutagenic or cytotoxic lesions such as 3′-azido and 2′,3′-didehydro nucleotides, which are significant in antiviral therapies. The enzyme’s efficacy in excising incorrect nucleotides inversely correlates with the gap size of the DNA, and its exonuclease function is critical in conjunction with DNA polymerase β to effectively repair and close nicks in the DNA strand [60].

Additionally, APEX1’s exonuclease activity is influenced by the canonicity of the nucleotide pairs and the chemical nature of the groups flanking these nicks [61]. Optimal conditions for this activity have been established to facilitate the use of photoreactive nucleotides for studying protein-DNA interactions through photoaffinity labeling. The presence of a hydroxyl group at the 5′ margin significantly enhances substrate specificity, favoring substrates with flapped or recessed ends [62]. Mutation studies reveal that changes in the hydrophobic pocket of APEX1, which aligns with similar pockets in related exonucleases, substantially modulate its substrate specificity and enzymatic efficiency, suggesting a potential regulatory mechanism in genomic stability maintenance through selective repair of 3′-mismatched nucleotides [63]. These findings underscore APEX1’s versatility and its critical role in maintaining genomic integrity against a backdrop of spontaneous and induced DNA damage.

### 2.4. Apex1 in Cardiovascular Physiology

Oxidative stress is a hallmark of cardiovascular diseases, driven by an imbalance between the production of reactive oxygen species (ROS) and antioxidant defenses. In the cardiovascular system, ROS are generated by endothelial cells (ECs), vascular smooth muscle cells (VSMCs), and cardiomyocytes, functioning as both signaling mediators and sources of cellular injury [64].

APEX1 is pivotal in mitigating ROS-induced damage through its dual role in DNA repair and redox regulation. As a core component of the base excision repair (BER) pathway [65], it corrects oxidative DNA lesions such as 8-oxoguanine and abasic sites [66]. Excessive accumulation of these lesions causes genomic instability, cell cycle arrest, and apoptosis, phenomena closely tied to atherosclerosis, myocardial infarction, and heart failure [67,68,69,70].

Additionally, APEX1’s redox function modulates key transcription factors relevant to cardiovascular physiology. For instance, APEX1 has been implicated in regulating NF-κB, a transcription factor central to inflammation and immune responses [50]. In situations of oxidative stress or exposure to proinflammatory cytokines, NF-κB promotes the expression of genes involved in cell proliferation and apoptosis [71,72], thus exacerbating endothelial dysfunction [73]. By controlling the redox state of NF-κB, APEX1 can curtail proinflammatory signaling and support vascular homeostasis [50]. This capacity to influence multiple redox-sensitive factors highlights APEX1’s broad significance in cardiovascular biology.

### 2.5. DNA Damage and Diseases of the Cardiovascular System

**Figure 3:**
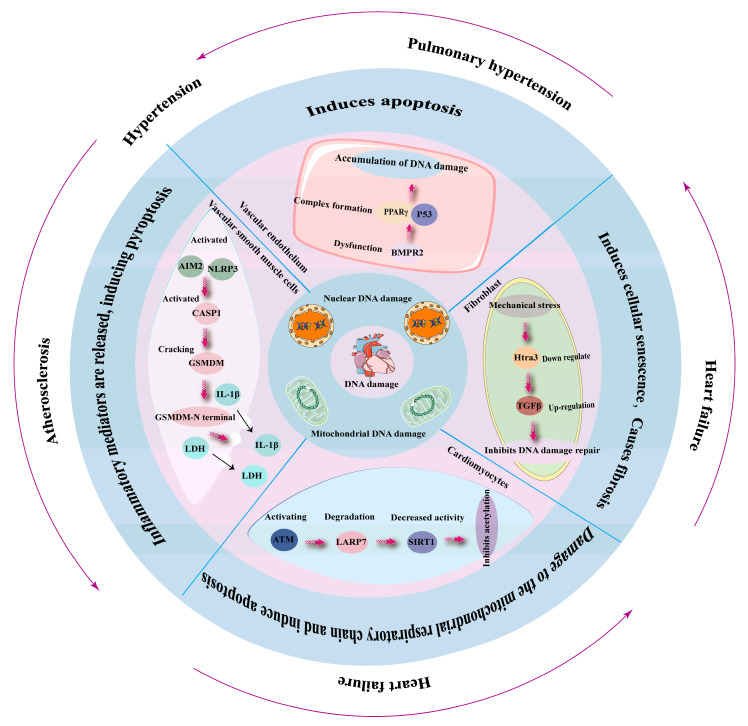
DNA damage and systemic diseases of the cardiovascular system. In the cardiovascular system, the accumulation of DNA damage in different cell types can lead to various cardiovascular diseases. For example, DNA damage accumulation in vascular smooth muscle cells and vascular endothelial cells can contribute to hypertension, pulmonary arterial hypertension, and atherosclerosis. Meanwhile, DNA damage accumulation in cardiomyocytes and cardiac fibroblasts can lead to myocardial fibrosis and heart failure.

DNA damage is a fundamental biological event that can lead to cellular dysfunction, aging, and various diseases, including those affecting the cardiovascular system (Figure 3) [74,75,76]. Oxidative stress, driven by excess reactive oxygen species (ROS), is a hallmark of cardiovascular diseases (CVDs). ROS can directly induce DNA lesions, such as single-strand breaks (SSBs) and base modifications like 8-oxo-2′-deoxyguanosine (8-oxo-dG) [77]. Persistent DNA damage activates the p53 pathway, exacerbating cellular senescence [78].

Atherosclerosis is strongly associated with DNA damage. Endothelial cells lining the arteries are constantly exposed to oxidative stress and inflammatory cytokines, leading to increased DNA damage [79]. Studies have demonstrated that oxidative DNA damage markers such as 8-hydroxy-2′-deoxyguanosine (8-OHdG) are elevated in atherosclerotic plaques [80]. DNA damage in endothelial cells induces senescence and apoptosis, promoting vascular dysfunction and plaque instability [81]. Moreover, deficiencies in DDR pathways, such as those involving ATM (ataxia-telangiectasia mutated) kinase and PARP (poly [ADP-ribose] polymerase), exacerbate atherosclerosis by impairing DNA repair [82]. Heart failure (HF) is a condition in which the heart fails to pump blood effectively. Myocardial cells are highly susceptible to oxidative stress, and prolonged exposure to ROS results in DNA damage accumulation [70]. Persistent DDR activation can trigger maladaptive responses, including cellular senescence and fibrosis, contributing to HF progression [83,84]. Furthermore, mutations in genes involved in DNA repair, such as XRCC1 and BRCA1, have been linked to increased susceptibility to HF [83,85]. Hypertension, a major risk factor for CVDs, has also been associated with DNA damage [86]. High blood pressure induces mechanical stress on vascular endothelial cells, leading to increased ROS production and subsequent DNA damage [86,87].

DNA damage plays a crucial role in the pathogenesis of CVDs, contributing to conditions such as atherosclerosis, heart failure, and hypertension. Defects in DNA repair mechanisms exacerbate these conditions, underscoring the importance of genomic integrity in maintaining cardiovascular health. Future research focusing on DDR modulation and targeted therapies may provide new avenues for preventing and treating CVDs.

## 3. APEX1 in Early Embryonic Development, Aging and Diseases

As a component of the DNA repair machine and redox-sensitive transcriptional cofactor for NF-κB, HIF-1, and AP-1 [88,89,90,91], APEX1 has a primary role in early embryonic development [92], tissue aging [93], and a wide spectrum of diseases, including cancers, neurodegenerative diseases, and cardiovascular diseases.

APEX1 is indispensable for proper embryonic development. Studies have shown that heterozygous Apex1 mutant mice develop into adulthood and are fertile without any gross abnormalities in size or behaviors [92,94,95,96]. In contrast, homozygous mutant mice die by embryonic day 5.5 or 9.5 [92,96]. Xanthoudakis et al. generated a null Apex1 allele lacking exons 1–4. Mice homozygous for this null allele are able to develop into normal blastocysts but turn necrotic at embryonic day 5.5 with disorganized patches of pyknotic cells [92]. Ludwig et al. built another null Apex1 allele by deleting exon 2 and part of exon 3. These Apex1-null mice fail to thrive between embryonic day 7.5 and 9.5, and the embryonic cell pyknosis is evident at embryonic day 6.5 [96]. Of note, Apex1-null embryos are sensitive to γ-irradiation, suggesting that BER defects are responsible for embryonic developmental failure [96].

On the other hand, both the DNA repair capacity and redox transcriptional functions of APEX1 have been indicated in the survival, proliferation, pluripotency, and differentiation of embryonic stem cells (ESCs). Intriguingly, APEX1 is a target gene of sirtuin 1 (SIRT1), a class III histone deacetylase that plays a primary role in the DNA repair process. The SIRT1-dependent expression of APEX1 synergizes with SIRT1 to maintain genomic stability and survival of human ESCs [97]. Proliferation of ESCs is also positively regulated by APEX1. Knockdown of APEX1 can reduce the cell number of E14 mouse ESCs via downregulation of cell cycle mediators and induction of G2/M phase arrest. Moreover, the pluripotency markers OCT4 (Octamer-binding transcription factor 4), NANOG, and SOX2 (SRY (sex determining region Y)-box 2) are also reduced upon APEX1 knockdown, which correlates with aberrant expression of neural differentiation markers NESTIN and MAP2 (Microtubule-associated protein 2). Such an alteration in stemness upon APEX1 knockdown is partially mediated by the suppressed expression of glial cell-derived neurotrophic factor (GDNF) and GFRα1, the cognate receptor for GDNF, as supplementation of GDNF rescues the proliferation, pluripotency, as well as teratoma formation by APEX1-depleted E14 ESCs. The functional role of APEX1 in ESCs is attributable to its redox function.

Domenis et al. reported a ROS-dependent APEX1-NFκB axis in the neuronal differentiation program of embryonic carcinoma cells NT2-D1 and human adipose tissue-derived multipotent stem cells [97]. Of note, the pro-neural effects of APEX1 in differentiating stem cells can be abrogated by E3330, a redox-selective APEX1 inhibitor [98]. Similarly, APEX1 has a crucial role in hemangioblast development from ESCs. Knockdown of APEX1 using siRNA reduces the frequency of blast colony-forming cells and the differentiation of erythroid progenitors and myeloid progenitors in cultured mouse ESCs [99]. These pro-hematopoietic effects of APEX1 are blocked by E3330 but not methoxyamine, an inhibitor of APEX1 endonuclease activity, suggesting the redox, but not DNA repair, function of APEX1 is critical for its roles in self-renewal and fate determination of ESCs.

Recently, an important study dove deep into the transcriptional role of APEX1 in cell fate transitions [100]. By recognizing DNA base modifications, APEX1 mediates negative DNA supercoiling to generate short, intense transcriptional bursts and amplify transcriptional noise, whereby it favors the neural ectodermal fate of ESCs and promotes fibroblast reprogramming of iPSCs [100]. These findings suggest that APEX1 is crucial for early embryonic development via both DNA repair and redox functions. However, due to early embryonic failure upon APEX1 knockout (KO), the roles of APEX1 in cell specification in vivo and organogenesis warrant further investigation using lineage-restricted KO models.

Several lines of evidence support the role of APEX1 in cellular senescence and organismal aging. First, Miriama et al. reported that the expression level of APEX1 decreases during the replicative aging of human ESCs, correlating with decreased BER efficiency and enhanced susceptibility to irradiation-induced DNA double-strand breaks (DSBs). Importantly, overexpression of APEX1 corrects this cellular aging-dependent DNA repair deficiency [6]. As unrepaired DNA damage is a well-established inducer of cellular senescence, this study suggests a senescence-regulatory role of APEX1, which was validated by a following seminal study that dissected the functional involvement of APEX1 in senescence [101]. In this study, Li et al. demonstrated the downregulation of APEX1 as primary human fibroblasts undergo replicative senescence, indicated by increased senescence-associated β galactosidase activity and increased expression of p16, p21, and γH2AX. It is interesting to note the accumulation of DNA damage at telomeres upon siRNA-mediated APEX1 knockdown, suggesting a telomeric DNA damage-driven senescence. Importantly, tamoxifen-induced conditional knockout of APEX1 in mice at postnatal days 7 to 12 leads to growth impairment and reduced size of multiple organs (heart, liver, brain, kidney, intestine, etc.) by postnatal day 28. Such a drastic whole-body phenotype is associated with elevated cellular senescence, but not apoptosis, in the skin and colon, especially the epithelial compartment. When APEX1 is ablated at 6 weeks of age or later, organ size is minimally altered, but premature aging characteristics, such as hair loss and impaired wound healing, are observed. Importantly, cellular senescence is still significantly increased in APEX1-null skin and colon.

Furthermore, in a spectrum of human tumors, including breast, thyroid, or glioblastoma malignancies, immunoreactivity of APEX1 in cancer tissues has a negative correlation with senescence, although there is no clarification on which tissue types, i.e., tumor, stroma, or immune cells, are senescent [101]. Despite the lack of a systematic report of APEX1 expression in the aging organs of humans, multiple animal studies have provided a rather complex picture of age-dependent alterations in APEX1 expression in a tissue- and subcellular localization-dependent manner. In mouse liver, the expression and activity of APEX1 do not vary between 4, 10, and 20 months of age. However, subcellular compartmentalization of APEX1 activity is changed in hepatocytes from old mice, with two- and six-fold increases in nucleus and mitochondria, respectively, and a two-fold decrease in cytoplasm [96]. In a rat cerebral cortex, an age-dependent decline in APEX1 activity was observed in isolated mitochondria, with cytoplasmic and nuclear activities remaining uninvestigated [102]. When comparing the immunoreactivities of APEX1 in kidneys from 6-, 24-, and 28-month-old male rats, senescence is found in proximal convoluted tubules in aged rats where decreased APEX1 levels are observed. However, APEX1 immunoreactivity was found to increase in distal convoluted tubules [103]. These findings indicate that APEX1 plays a critical role in regulating cellular senescence and organismal aging, but its tissue-specific roles warrant further careful study. Another noticeable future direction is to distinguish the mechanistic roles of APEX1 in chronological aging versus biological aging. This will require efforts to interrogate the causal involvement of APEX1 in human diseases or animal models featuring premature aging.

Emerging evidence has established the important roles of APEX1 in a diverse collection of diseases. In terms of the relevance of APEX1 to cancers and neurodegenerative diseases, we refer readers to recent excellent reviews [1,104]. Here, we briefly review the evidence on APEX1 and inflammatory diseases, using the liver as a model system. APEX1 and specific cardiovascular diseases will be discussed in the following sections.

APEX1 has pronounced roles in both innate and adaptive immunity. ROS activates APEX1 expression in immune cells (macrophages and lymphocytes) and parenchymal cells, whereby it mediates the expression and release of cytokines and chemokines, likely via its redox regulation of the transcriptional activities of NF-κB, AP-1, p53, and STAT3. For example, Zhang et al. reported the early induction of APEX1, most strongly in hepatocytes as well as peri-portal immune cells to a lesser extent, during acute liver injury following rat liver transplantation [105]. The proposed mechanism involves ischemia-reperfusion injury, a complication of liver transplantation, which generates a wealth of ROS inflicting DNA damage, in turn leading to the activation of poly(ADP-ribose) polymerase, accumulation of p53 and lipid peroxidation, thereby increasing calcium levels [106]. Elevated APEX1 abundance is reported to facilitate the chromatin binding of p53 [54]. Furthermore, Apex1 heterozygous KO (Apex1^+/−^) mice exhibit heightened susceptibility to 2-nitropropane-induced oxidative DNA damage in the liver, associated with increased cell apoptosis and p53 protein abundance [107], implicating a pro-apoptotic role of the APEX1-p53 axis in hepatocytes under oxidative stress.

Moreover, the APEX1-NF-κB cascade plays a significant role in tissue inflammation. Upon liver injury, the chromatin binding of NF-κB is rapidly enhanced, prompting the expression of inflammatory mediators such as IL-1, tumor necrosis factor α (TNF-α), and intercellular cell adhesion molecule-1 (ICAM-1), thereby exacerbating liver inflammation [108,109,110]. In the 2-nitropropane-injured liver, the NF-κB DNA binding activity is dampened in Apex1^+/−^ mice [107], associated with reduced hepatocyte apoptosis and liver injury as assessed by reduced serum aspartate aminotransferase level. Even more intriguingly, APEX1 can mitigate the acute rise in superoxide production by 5 min post-reperfusion of the liver. This antioxidant effect is attributed to the physical interaction between APEX1 and Rac1 (Ras-related C3 botulinum toxin substrate 1), which suppresses Rac1 activity and subsequent ROS generation [111]. Recently, Sawakami et al. performed an unbiased transcriptomic analysis of the peripheral blood genes related to immune tolerance following liver transplantation and identified APEX1 as one of the top expressed genes in patients with tolerance [112]. Manipulation of APEX1 expression in human fetal hepatocyte line L-02 cells showed that APEX1 suppresses the release of multiple cytokines, including TNF-α, IFNγ, IL-1β, and IL-10. ShRNA knockdown of APEX1 doubles the apoptotic rate of L-02 cells. In addition, polymorphism of the Apex1 gene (rs176094, rs1130409), together with other BER genes (LIG3 rs4796030 and NEIL1 rs4462560), has been associated with the risk of non-alcoholic fatty liver disease [113]. Taken together, these findings suggest an anti-inflammatory role of APEX1 in the context of non-tumor liver diseases. Nevertheless, the relationships between APEX1 and inflammation have been extensively studied in a variety of organ systems and diseases, for which Oliveira et al. recently published an excellent review [47].

## 4. APEX1 in Cardiovascular Cells and Diseases

**Figure 4:**
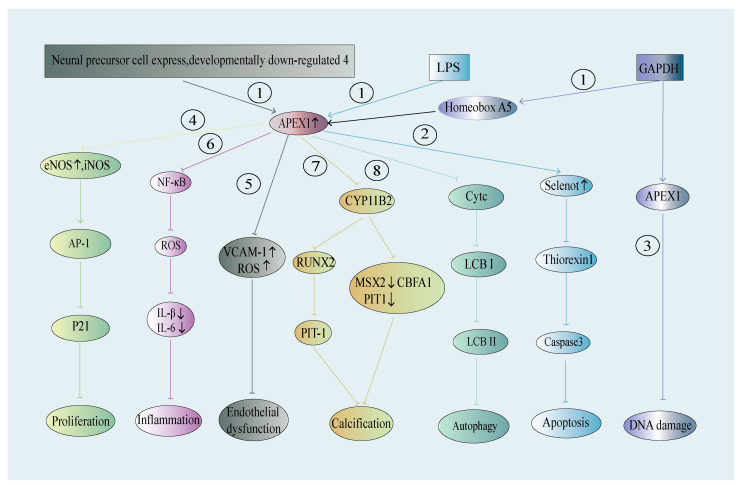
Regulation of APEX1 Expression Through Different Mechanisms and the Function of APEX1 in cardiovascular pathophysiology. (1) Activation of APEX1 by various factors, resulting in the gene transcription of Apex1; (2) Mechanism of APEX1 antiapoptotic function; (3) Mechanism of APEX1 DNA damage repair; (4) Mechanism by which APEX1 inhibits proliferation; (5) Mechanism by which APEX1 protects vascular endothelial function; (6) Mechanism of APEX1 anti-inflammatory function; (7) Mechanism of APEX1 anticalcification; (8) Mechanism of APEX1 antiautophagy.(↑: Increase the content of this component in the cells; ↓: Decrease the content of this component in the cells).

APEX1 regulates its effects on the cardiovascular system primarily by modulating a wide spectrum of functional behaviors, including proliferation [114], apoptosis [115], inflammation [116], autophagy [50], and phenotypic plasticity [117] of endothelial cells (ECs), smooth muscle cells (SMCs), and cardiomyocytes (Figure 4). Emerging evidence from human genetics, specimens and animal models indicates a significant role of APEX1 in a panel of cardiovascular diseases, including hypertension, restenosis, atherosclerosis, coronary artery disease, and stroke. Aggarwal et al. integrated protein-protein interaction (PPI) and protein translational modification (PTM) results from several databases, identifying APEX1 as a potential therapeutic target for cardiovascular diseases [71]. Importantly, many aspects of APEX1 functions can be explained by its direct effects on the functional behaviors of ECs, SMCs, and cardiomyocytes (Table 1). Here, we will review and discuss the important data pertaining to the involvement of APEX1 in cell biology and pathophysiology in the context of cardiovascular diseases.

### 4.1. APEX1 in ECs—Its Role in Hypertension, Atherosclerosis, and Other Vascular Diseases

Evidence suggests that APEX1 in blood ECs exerts its regulatory influence on the progression of hypertension and atherosclerosis. Various pathogenic factors associated with hypertension and atherosclerosis can modulate the expression and/or activity of APEX1 in human vascular ECs, including ROS, shear stress, TNF-α, LPS, hypoxia, and oxidized low-density lipoprotein (ox-LDL).

#### 4.1.1. APEX1 in ECs and Hypertension

Hypertension is a prevalent, chronic disease and a significant risk factor for cardiovascular diseases. It affects approximately 20% of individuals in developed countries, such as Europe and America, and it is increasing annually. The causes and mechanisms of hypertension are complex. However, it is recognized that the renin-angiotensin-aldosterone system plays an essential role in the pathogenesis of hypertension [118], and angiotensin receptor blockers are one of the common prescription drugs for hypertensive patients. Mechanistically, renin catalyzes the conversion of angiotensinogen into angiotensin I, which is further cleaved by angiotensin-converting enzyme into angiotensin II. Notably, angiotensin II can stimulate the release of aldosterone from the adrenal cortex. In addition to regulating sodium and water reabsorption in the renal tubular system, both angiotensin II and aldosterone have direct effects on cardiomyocytes and vascular wall-resident cells, including ECs, vascular SMCs and fibroblasts, contributing to the development of hypertension [118]. Moreover, recent research suggests that inflammation, endothelial dysfunction [119] and vascular aging may play a detrimental role in hypertensive disease and its complications [120,121,122,123,124,125].

The APEX1 polymorphism has been associated with essential hypertension [126]. In a case-control study involving 265 patients with essential hypertension, a combined APEX1 rs1760944-rs3136817-rs1130409 single nucleotide polymorphism (SNP) showed differential distribution between hypertensive patients and normotensive controls, with a specific G-T-T haplotype having a statistically higher prevalence in hypertensive patients. Moreover, a multivariate logistic regression analysis supported G-T-T diplotype/heterozygote as a risk predictor for hypertension, independent of traditional risk factors, including body mass index, total cholesterol, hyperlipidemia, and diabetes.

Mechanistic insights from animal models and cell culture studies have indicated a critical role of APEX1 in endothelium and hypertensive pathogenesis. An early study by Jeon et al. employed APEX1^+/−^ mice to establish a causal role of APEX1 in the prevention of endothelial dysfunction and subsequent hypertensive phenotypes [127]. Reduced plasma nitric oxide (NO), impaired endothelium-dependent vasodilation of isolated aortic rings, and an approximately 40 mmHg increase in mean arterial blood pressure are observed in young adult APEX1^+/−^ mice. Conversely, adenoviral delivery of APEX1 into ex vivo cultured rat aortas, which appears to primarily increase APEX1 protein abundance in endothelium, improves endothelium-dependent vasodilation. This endothelial benefit of APEX1 is attributable to its activation of the PI3K-AKT axis and subsequent activation of eNOS and NO production in a redox function- and nuclear localization-dependent manner. Several studies have investigated how APEX1 stimulates the PI3K-AKT cascade in ECs. H-Ras is a potential target regulated by APEX1, as its total protein expression and active, the GTP-bound form is elevated by APEX1 overexpression in human umbilical vein endothelial cells (HUVECs) and downregulated in APEX1^+/−^ mouse aortas [127]. Interestingly, although the redox function, specifically cysteine (Cys)65/93 residues, of APEX1 is required for its effects on H-Ras and eNOS activation, ROS levels in APEX^+/−^ mice aortic wall remains unaltered [127]. Of note, SIRT1, a class III histone deacetylase (HDAC), was identified as a direct downstream target of APEX1 [128]. Through the Cys65/93-dependent redox function, APEX1 reduces Cys371/374 of SIRT1 to maintain its deacetylase activity on eNOS. Functionally, adenoviral overexpression of SIRT1 rescues the dampened endothelium-dependent vasodilation of isolated APEX1^+/−^ aortas associated with restored NO production [128]. In addition, Lee et al. reported that APEX1 can inhibit the serine phosphorylation of SHC adaptor protein 1 (p66shc) mediated by protein kinase C beta II (PKCβ II), block the activation of p66shc triggered by ox-LDL in ECs, and alleviate vascular constriction caused by PKC activation [129].

It should be noted that none of the studies above were performed using animal models with endothelium-specific inactivation of APEX1. As APEX1 is a ubiquitously expressed protein in a wide spectrum of organs and cell types, it is entirely possible that APEX1 in non-endothelial tissues contributed to the suppression of hypertensive phenotypes. Critically, in the presence of L-NAME, an NO synthase inhibitor, APEX1^+/−^ aortas exhibit much lower force generation in response to phenylephrine [127], a vasoconstrictive stimulus, suggesting endothelium-derived NO may not be the only vascular defect upon APEX1 haploinsufficiency that contributes to dysregulated arterial tone and blood pressure regulation. Moreover, the APEX1-SIRT1 axis is functional in the heart, liver, and kidney, in addition to large arteries [128]. Indeed, Sengupta et al. showed that, in As4.1 mouse renal epithelial cells, APEX1 forms a transcriptional corepressor complex with HDAC1 on the enhancer of the REN gene, which encodes renin [130]. APEX1^+/−^ mice exhibit elevated renin transcript levels in the kidney and renin activity (i.e., angiotensin I production) in plasma [130]. These findings provide evidence that non-endothelial APEX1 regulates blood pressure.

In summary, existing research suggests that APEX1, in endothelial and/or renal epithelial compartments, plays a role in lowering blood pressure. The relative contributions of endothelial versus non-endothelial APEX1 in hypertensive phenotypes require further studies using animal models with tissue-specific inactivation of APEX1. Augmenting APEX1 expression or activity may contribute to alleviating the pathogenesis of hypertension. However, although the chemical activator of APEX1 endonuclease has been reported [131], the small-molecule activator of APEX1 redox signaling remains unidentified. Therefore, genetic or pharmacologic approaches to targeting APEX1 in vivo warrant further investigation.

#### 4.1.2. APEX1 in ECs and Atherosclerosis

As one of the primary causes of mortality, atherosclerosis is a prevalent disease that poses a significant threat to human health. The incidence of this disease has been steadily rising across different age groups [132,133,134]. Atherosclerosis is characterized by the formation of plaques on the inner surface of blood vessels (most commonly the large arteries), leading to narrowing or even occlusion of the vascular lumen. The lack of blood flow can lead to tissue ischemia. Even more devastating, thrombotic or embolic events due to plaque instability cause catastrophic events in end-organs, such as myocardial infarction or stroke. Atherogenesis follows a complex, multi-stage course. The initial phase is EC injury and consequent endothelial dysfunction, which can be induced by a variety of atherogenic risk factors, including hypertension, diabetes, smoking, and hyperlipidemia. The compromised endothelium has a weakened barrier function and expresses pro-inflammatory adhesive molecules and chemokines. The former facilitates the accumulation of LDLs at the subendothelial space. The latter facilitates the adhesion and infiltration of immune cells, which creates a persistent inflammatory microenvironment that activates vascular wall resident cells, including ECs, SMCs, and fibroblasts.

During the early stage of atherogenesis, SMCs migrate from the media to the intima, while subsets undergo clonal expansion, constituting the major cellular mass of the intima. Subsequently, both infiltrated macrophages and activated SMCs contribute to the formation of lipid-laden foam cells. Simultaneously, SMCs on the luminal side deposit collagenous extracellular matrix over the lipid core, forming a fibrous cap that is essential to the structural integrity of atherosclerotic plaque. However, uncontrolled inflammatory conditions can cause the fibrous cap to destabilize due to reduced SMC vitality and reduced collagen synthesis, which leads to plaque rupture and thrombus formation, which are the main triggers of cardiovascular events [135,136]. A variety of cellular stress signals that exacerbate the development of atherosclerosis have been proposed, including lipid peroxidation damage, endothelial dysfunction, SMC phenotypic dysregulation, chronic inflammation, iron overload, and notably oxidative stress and consequent DNA damage [137,138,139].

APEX1 expression has been detected in all the major cell types, including ECs, SMCs and macrophages in mouse or human atherosclerotic lesions [140,141,142]. Notably, Lee et al. demonstrated an increase in APEX1 expression in the atherosclerotic thoracic aortas of ApoE^−/−^ mice fed a Western diet [142]. Although the high-resolution colocalization of APEX1 with specific cell labels is lacking in this study, the elevated immunoreactivity of APEX1 in the endothelium, medial wall, and lesional leukocytes is clearly discernible.

Emerging evidence supports the functional role of APEX1 in endothelial inflammation. Kim et al. discovered that APEX1 could suppress the adhesion of ECs to monocytes, a process induced by TNF-α and executed via surface presentation of a panel of cell adhesion molecules in ECs, such as vascular cell adhesion molecule-1 (VCAM-1), ICAM-1, and E-selectin. This activation leads to the recruitment and migration of monocytes and neutrophils to the subendothelial layer [143]. The inhibitory effects of APEX1 on leukocyte-endothelial adhesion are primarily mediated by a NOS-dependent mechanism, as L-NAME suppresses the effect of APEX1 on endothelial expression of VCAM-1. Of note, overexpression of APEX1 also diminishes TNF-α-induced superoxide production and p38 MAPK activity [143]. These properties of APEX1 suggest that its presence in ECs may inhibit this initial stage of atherogenesis. Moreover, transplantation of APEX1-overexpressing endothelial progenitor cells leads to a two-fold increase in homing/adhesion to the wire-injured mouse femoral artery, associated with a 60% reduction in neointima formation [144]. This effect is likely related to the redox signaling of APEX1 without altering overall ROS production, as E3330, an inhibitor of APEX1 redox function, reduced the in vitro adhesion of endothelial progenitor cells to fibronectin-coated substratum. This line of evidence implicates the role of APEX1 in the maintenance of endothelial integrity to quench intimal hyperplasia, an early event of atherosclerosis.

The N-terminus of APEX1 is critical to its protective effects against EC inflammation and apoptosis. Merk et al. conducted a bulk RNA sequencing of primary human ECs infected with a lentiviral vector expressing the first 20 amino acids of APEX1 or an empty vector and treated with 150 ng/mL of active or detoxified LPS [145]. Differential expression analysis and gene set enrichment analysis (GSEA) demonstrated that while APEX1 overexpression has no overall impact on the EC transcriptomes at baseline, it significantly diminishes TNF-α signals in LPS-treated ECs. PXDN, which encodes vascular peroxidase 1, and SELENOT, which encodes selenoprotein T, are among the most significantly upregulated genes by APEX1 in LPS-challenged ECs, and selenoprotein T likely mediates the protective effects of APEX1 against EC inflammation and apoptosis.

The anti-inflammatory effects of APEX1 have gained support from other independent studies. Xu et al. found that APEX1 antagonizes ox-LDL-induced EC inflammation and dysfunction (i.e., reduced NO production) in HUVECs [146]. Interestingly, the expression of APEX1 in ECs is controlled by neuronally expressed developmentally downregulated protein 4 (NEDD4), which protects against vascular calcification. The level of NEDD4 is downregulated by ox-LDL in ECs, correlated with increased apoptosis and reduced eNOS activity. siRNA knockdown of APEX1 abolishes the endothelial benefits of NEDD4 overexpression [146]. Moreover, APEX1 has been shown to counteract EC apoptosis induced by multiple factors. Angkeow et al. discovered that overexpression of APEX1 inhibits hypoxia/reoxygenation (H/R) and TNF-α-induced cell apoptosis [50]. However, it was found to be ineffective against oxidative stress-induced cell apoptosis caused by H_2_O_2_. This indicates that APEX1 does not neutralize H_2_O_2_ by enhancing the expression of antioxidant defenses, such as catalase and glutathione peroxidase. Instead, it inhibits the production of H_2_O_2_ within cells to resist H/R-induced EC apoptosis. Additional mechanistic studies revealed that overexpression of APEX1 inhibits the nuclear translocation of p50 and p65 stimulated by H/R, indicating that APEX1 inhibits the NF-kB signals triggered by H/R stimulation [73]. Hall et al. found that overexpression of APEX1 inhibits hypoxia- and TNF-α-induced EC apoptosis through both the NF-kB-independent and -dependent pathways and identified the apoptotic functional region of APEX1 within the redox region [50].

APEX1 also contributes to mitochondrial protection, angiogenesis, and regulation of cell ion channels in ECs. Joo et al. found that mitochondrial APEX1 can inhibit PKC-induced mitochondrial dysfunction in mouse ECs [147]. PMA, an activator of PKC, prompts mitochondrial depolarization and clusters of ROS while also promoting APEX1 mitochondrial translocation. Further investigations revealed that overexpression of APEX1 inhibits PMA-induced mitochondrial dysfunction while silencing of the APEX1 gene exacerbates mitochondrial dysfunction [147]. In an independent study, Luo et al. suggested that mouse retinal vascular endothelial cell (RVEC) capillary formation is highly sensitive to redox inhibition of APEX1 [148].

Disturbed blood flow is an important atheroprone signal that elevates ROS production and inflammatory signaling in ECs. Intriguingly, APEX1 has been identified as an endothelial mechanotransducer in response to oscillatory shear stress, which causes the nuclear translocation of APEX1 [149]. Oscillatory shear or TNF-α promotes the binding of p300 acetyltransferase to APEX1, and the acetylation facilitates its nuclear enrichment, enabling its transcriptional regulation of inflammatory and atherogenic programs. Specifically, shear-activated APEX1 reciprocally orchestrates endothelial NF-κB signaling via physical interaction with p65 and p50 subunits. siRNA knockdown of APEX1 in primary HUVECs reduced the expression of VCAM-1, ICAM-1, and E-selectin induced by oscillatory flow or TNF-α. Importantly, a mouse model with tamoxifen-inducible, endothelium-specific APEX1 KO (Cdh5-CreERT2; Apex1^flox/flox^) was developed in this study. The atherosclerotic lesions at carotid arteries are induced by AAV9-PCSK9 and partial carotid ligation. It is very intriguing to note that endothelial APEX1 KO almost abrogated the formation of atheroma by 4 weeks post-ligation, and this atheroprotective effect was potentially acting at the early stage, as a >90% reduction in intima-to-media ratio and almost complete lack of F4/80+ macrophages in the arterial wall were observed. Of note, these recent powerful findings are in critical opposition to a previously suggested vasoprotective role of APEX1 in the endothelium. However, further efforts are warranted to elucidate the mechanotransducive transcriptomics orchestrated by APEX1 in endothelium and atherogenesis.

Taken together, current evidence presents both protective and detrimental roles of APEX1 in ECs throughout the early stages of atherogenesis, including endothelial inflammation and intimal hyperplasia. However, several critical points need to be addressed to achieve a better understanding of endothelial APEX1 in atherosclerosis. First, the animal study with endothelium-restricted inactivation of APEX1 needs to be repeated using different models of atherogenesis and with extended coverage of earlier and later time points, in addition to 4 weeks of partial carotid ligation. Second, the molecular cascade that APEX1 exerts its positive or negative effects on endothelium remains unclear. For instance, how is APEX1 involved in endothelial shear sensing and what are the transcriptional events, in addition to NF-κB, targeted by APEX1 to suppress endothelial inflammatory signals? Third, although evidence supports the importance of the N-terminal redox function of APEX1 in ECs and atherosclerosis, does the C-terminal endonuclease activity, i.e., DNA repair capacity, of APEX1 play a role in atherosclerosis? In this regard, it is important to note that DNA damage is a major determinant of vascular aging and EC senescence [150].

#### 4.1.3. APEX1 and Stroke—Potential Involvement of EC APEX1

Evidence from human genetics and animal models supports the role of APEX1 in stroke. In a case-controlled study involving 177 patients with cerebral infarction and 309 control subjects, a combined APEX1 rs1760944-rs3136814-rs1130409 SNP was differentially distributed, with a higher prevalence of the G-C-T haplotype in both male and female patients with cerebral infarction [151]. In a rat model of diabetic stroke, E3330, a small-molecule inhibitor of APEX1 redox activity, improves the recovery of neurological functions and the integrity of the blood–brain barrier (BBB), suggesting a possible role of endothelial APEX1 in post-stroke recovery [152]. In line with this finding, siRNA against APEX1 enhances the barrier functions, as assessed by transendothelial electrical resistance, of HULEC-5a human lung microvascular ECs treated with an HSP90 inhibitor AUY-922 [153]. However, the dynamics of stroke involve a complex interplay of different types of brain cells, including neural cells, immune cells, and vascular cells. Even vascular cells that comprise the BBB include ECs and pericytes. All these cells can be targeted by E3330 in the context of a compromised BBB during stroke. In fact, there is good evidence that supports the regulatory role of non-endothelial APEX1 in stroke. For instance, oligomycin, a mitochondrial ATP synthase inhibitor, or H_2_O_2_ treatment causes the downregulation of nuclear abundance of APEX1, but not the BER proteins LIG3 and XRCC1, in primary mouse cortical neurons, implicating a neuroprotective role of APEX1 [153]. Also, glucagon-like peptide 1 (GLP-1) promotes DNA repair in primary rat cortical neurons under oxidative stress induced by menadione, an inhibitor of the mitochondrial electron transportation chain. This genome-stabilizing effect is at least partially via APEX1, as APEX1 is the only BER protein that is activated by GLP-1 [154]. Of note, to bypass the embryonic death of APEX1 KO, mice with tamoxifen-inducible APEX1 KO (CAGGCre-ER; Apex1^flox/flox^) have been developed [155]. Induction of global APEX1 KO in adult mice 3–4 months of age does not lead to a quick death, although BER activity in the brain is abolished. Upon transient focal cerebral ischemia, APEX1 KO mice have aggravated stroke phenotypes, as compared with wild-type control mice, including significantly higher mortality, larger brain infarct volume, impaired neurological functional recovery, and enhanced neuronal DNA damage, apoptosis, degeneration, and loss [155]. Unfortunately, the dynamics of cerebral blood flow restoration and microvascular reconstruction following stroke were not studied in these mice. Taken together, APEX1 may possess cell type-specific roles in post-stroke recovery, i.e., barrier-disruptive in the endothelium and pro-survival in neurons, which warrant further studies using tissue-specific APEX1 KO.

**Table 1:** Expression of APEX1 in Cardiac and Vascular Cells/Tissues.

Cell Type/Tissue		Agents/Models/Samples	Expression Levels	Effects of APEX1 Expression
Cardiomyocytes	H9c2/Hips-CMs cells	Oxidative stress (H_2_O_2_)	Protein ↓	Involved in apoptosis via caspase 3 pathway [156]
	Heart samples (mice)	Transverse aortic constriction (TCA)	WT TCA: Protein ↓ Dec1 KO TAC: Protein ↓ Dec1 KO: Protein →	N/A [157]
	Neonatal cardiomyocyte (rat)	Miconazole-stimulated	mRNA ↓, Protein ↓	Cell apoptosis [158]
	H9c2 (rat)	Hypoxia/reoxygenation (H/R)	Protein ↑	Overexpression of APE1 attenuates cardiac H/R injury and promotes PINK1/Parkin-mediated mitophagy in H/R-injured H9c2 cells [159]
Endothelial Cells (ECs)	HUVECs	Oxidized low-density lipoprotein	Protein ↓	Inflammation and dysfunction [146]
	MS-1 (CRL-2279™) (mice)	phorbol 12-myristate 13-acetate (PMA)	Mitochondrial: Protein ↑ Nucleus: Protein ↓	Mitochondrial dysfunction [147]
	HUVEC, HEK 293, liver/kidney/ aorta samples of mice	Oxidative stress (H_2_O_2_) APE1/Ref-1^+/−^ mice	Protein ↓	Endothelial SIRT1 inactivation [128]
	BPAEC/HULEC-5a	17-AAG, 17-DMAG, AUY-922	Protein ↓	Enhances the barrier effect in HULEC-5a and BPAEC [160]
	HUVECs, CPAEs	Hypoxic injury, TNF-α	Protein ↓	HUVECs and CPAEs apoptosis [50]
	HUVECs	TNF-α	Protein ↓	Monocyte adhesion in ECs [143]
	Primary human ECs	Oxidative stress (H_2_O_2_)	Protein ↓	Apoptosis [161]
Vascular Smooth Muscle Cells (SMCs)	Vascular SMCs (rat)	Phosphate-induced	Protein ↓	Apoptosis, calcification, and osteoblastic phenotype changes in vascular SMCs [117]
	Rat aortic SMCs	H_2_O_2_	Protein ↓	DNA damage, apoptosis and cell death [115]
	Vascular SMCs (mice)	iNOS KO	N/A	Altered AP-1/Ref-1 redox pathway and reduced proliferative response [114]
	RASMC, Rat carotid arteries	Angiotensin II, Balloon-injured	Nuclear fraction: Protein ↑ Cytosolic fraction: Protein ↓ Total →	Neointimal formation and vascular SMC migration [162]

Annotation: ↓: mRNA and protein levels downregulated; ↑: mRNA and protein levels upregulated; →: mRNA and protein levels unchanged.

### 4.2. APEX1 in Vascular SMCs—A Role in Arterial Neointima and Calcification

APEX1 has direct effects on the proliferation, apoptosis, and motility of vascular SMCs, with common and distinct signaling mechanisms from ECs. For example, an antisense oligonucleotide against APEX1 reduces PDGF-BB-induced thymidine incorporation and S-phase entry by 50% in primary rat aortic SMCs [163], and this pro-proliferative effect is executed through modulation of chromatin binding of AP-1. A similar APEX1-AP-1 axis may also explain the reduced proliferation and cell cycle progression in primary aortic SMCs isolated from iNOS-KO mice [163]. Interestingly, iNOS-null SMCs display reduced protein abundance of APEX1 in both cytoplasmic and nuclear fractions, with a more severe loss in nuclei, suggesting both expression and subcellular localization of APEX1 are regulatory components of SMC proliferation.

The antiapoptotic role of APEX1 in SMCs is mediated by physical interaction with GAPDH in a redox-sensitive manner. The nuclear GAPDH-APEX1 complex preserves APEX1 activity and protects SMCs against oxidative stress-induced DNA damage and apoptosis. In addition, GAPDH also enhances HOXA5-dependent transcription of the APEX1 gene. Interestingly, GAPDH expression is reduced in SMCs residing in the atherosclerotic plaque fibrous cap compared with the healthy aortic arch wall of ApoE^−/−^ mice [115].

APEX1 in vascular SMCs is reported to have a functional role in neointima formation within injured arteries. For example, APEX1 mediates angiotensin II- or H_2_O_2_-induced expression of sphingosine-1-phosphate receptor 1 (S1PR1), in turn promoting rat aortic SMC migration and contributing to neointima formation in balloon-injured rat carotid artery [162]. Upon angiotensin II-mediated nuclear translocation, APEX1 directly binds to the promoter of S1PR1, which is likely responsible for the upregulation of S1PR1 expression. Evidence also supports other cellular mechanisms through which APEX1 promotes intimal hyperplasia [162]. Basi and Adhikari et al. reported that APEX1^+/−^ mice displayed a 50% reduction in the neointima-to-media ratio in wire-injured femoral arteries. There is no difference in cell proliferation (BrdU labeling) or apoptosis (TUNEL) in the injured arteries between wild-type and APEX1^+/−^ mice. Instead, the induction of IL-1α, IL-10 and VEGF-A (Vascular Endothelial Growth Factor A) is largely impaired in APEX1^+/−^ arterial wall.

Although the nuclear immunoreactivity of the NF-κB p65 subunit in vascular cells remains similar, the in vivo transcriptional activities, e.g., the occupancy on target gene promoters of NF-κB, remain unaddressed. Similarly, the involvement of other APEX1-related transcription factors, such as AP-1, HIF-1α, and p53 [164], requires further studies. On the other hand, there is evidence supporting an opposite, antineointima role of APEX1 in SMCs. Lee et al. showed that adenoviral overexpression of APEX1 impairs PDGF-BB-stimulated migration of primary rat aortic SMCs, likely through inhibition of Syk (Spleen Tyrosine Kinase) kinase activity, and the downstream activity of p38 MAPK (Mitogen-Activated Protein Kinase), ERK1/2 (Extracellular Signal-Regulated Kinases 1 and 2), and HSP27 (Heat Shock Protein 27). This effect of APEX1 acts at the level of phosphorylation and activation of PDGFR (Platelet-Derived Growth Factor Receptor) β, and the underlying molecular mechanisms include dampened Rac1 activity and superoxide production by ectopic expression of APEX1. Importantly, the delivery of adenovirus encoding wild-type, but not redox mutant, APEX1 reduced intimal hyperplasia in the balloon-injured carotid artery in rats [162]. It remains unclear how to interpret the conflicting roles of APEX1 in different studies. In addition, considering none of the studies above manipulate APEX1 expression in SMCs specifically, it is entirely possible that APEX1 in ECs, SMCs, or leukocytes may exert variable functions on intimal hyperplasia.

Vascular calcification is characterized by abnormal calcium deposition in the vascular wall, which is primarily attributed to the aberrant osteogenic differentiation of vascular SMCs. Of note, calcification of the arterial wall is a prominent feature of atherosclerosis and vascular aging [165,166]. Lee et al. demonstrated that APEX1 expression is suppressed by inorganic phosphate (a metabolic stressor for calcification) in primary rat aortic SMCs [117]. Adenovirus-mediated overexpression of APEX1 inhibits phosphate-induced ROS production, apoptosis, and calcification of vascular SMCs and ex vivo cultured rat aorta. Such protective effects are associated with a contractile-to-osteogenic phenotypic transition of SMCs, and APEX1 is found to prevent phosphate-induced expression of osteogenic transcription factors RUNX2 (Runt-related transcription factor 2) and PIT-1 (Pituitary-specific positive transcription factor 1). Importantly, a redox mutant (C65A/C93A) of APEX1 loses the anticalcific effects in vascular SMCs, implying the involvement of the redox function of APEX1 [117].

As both intimal hyperplasia and vascular calcification are important pathogenic features of atherosclerosis, current evidence suggests a functional role of SMC APEX1 during atherogenesis. However, future studies using SMC-specific APEX1 KO animals would be required to examine this intriguing possibility.

### 4.3. APEX1 in Cardiomyocytes—A Role in Myocardial Ischemia Injury

In general, APEX1 has an antiapoptotic role in cardiomyocytes, mostly based on evidence derived from cell culture studies. Two independent groups identified APEX1 as a cytoprotective protein against cardiotoxicity induced by miconazole, an antifungal drug that elevates superoxide production and apoptosis in cardiomyocytes [156,158]. Won et al. performed an unbiased microarray screening using miconazole-treated rat neonatal cardiomyocytes in culture [158]. APEX1 is among the top miconazole-downregulated genes pertaining to redox homeostasis, and adenoviral overexpression of APEX1 almost completely abrogates ROS generation and apoptosis by miconazole. Moreover, miconazole-sequestered spontaneous cardiomyocyte contraction is also rescued by APEX1, suggesting a role of APEX1 in cardiac muscle bioenergetics and contractile functions. Similar antioxidant and antiapoptotic roles of APEX1 are supported by additional studies using H9c2 embryonic rat cardiomyocyte-like cell line challenged with miconazole [156]. Mechanistically, the BER activity of APEX1, especially on mitochondrial DNA repair, underlies its cytoprotective effects on cardiomyocytes [167]. The protective role of APEX1 in cardiomyocytes is further consolidated by Zhang et al. [168]. Using integrated transcriptomics and high-throughput transcription activity analysis of H9c2 cells treated with H_2_O_2_, APEX1 was identified as a sensitive transcription factor in response to oxidative damage in cardiomyocytes. Specifically, the transcriptional activity and nuclear abundance of APEX1 are downregulated by H_2_O_2_ in both H9c2 cells and iPSC-derived cardiomyocytes.

Recently, Tang et al. demonstrated a highly intriguing stimulatory role of APEX1 in PINK1/Parkin-mediated mitophagy, a select autophagy that removes damaged mitochondria, which correlates with the prevention of apoptosis of H9c2 cells [159]. This pro-mitophagic effect may reflect the effects of APEX1 on the generic autophagy machine, as APEX1 overexpression elevates the LC3B-II/-I protein ratio, which is an essential component of autophagosome biogenesis and reduces the protein abundance of p62, an autophagic cargo protein, suggesting that APEX1 promotes autophagic flux in cardiomyocytes. Moreover, APEX1, but not the redox-deficient mutant C65A, suppresses endoplasmic reticulum (ER) stress to counteract the apoptosis of H9c2 cells induced by palmitic acid [169]. Although the molecular cascades for APEX1 regulation of autophagy and ER stress remain elusive, the APEX1 axis may accelerate the elimination of oxidative damaged organelles (mitochondria or ER), which, in combination with its BER activity, maintain cardiomyocyte vitality under oxidative stress [159].

The APEX1 protein level is increased in cultured cardiomyocytes injured by H/R [159]. A careful examination of in vivo APEX1 expression in rat myocardium revealed that ischemia-reperfusion injury leads to an increase in cytosolic APEX1 abundance and a decrease in nuclear abundance in rat left ventricular wall [170]. Such a fluctuation in APEX1 expression is normalized by ischemic preconditioning, a protective measure against myocardial ischemia-reperfusion injury. Notably, intraventricular injection with antisense oligonucleotide against APEX1 abolishes the benefits of preconditioning on myocardial injury, as assessed by cardiac function, infarct size, and cardiomyocyte apoptosis. The molecular mechanism of cardioprotective effects of APEX1 involves its redox regulation of NF-κB and NRF2 (Nuclear Factor Erythroid 2–Related Factor 2) via physical interaction in nuclei. Of note, the co-localization between APEX1 and the NF-κB p50 subunit was carefully analyzed in cardiomyocyte nuclei using high-resolution immunofluorescence-confocal microscopy [170]. In addition, Jin et al. measured serum APEX1/Ref-1 levels in BALB/c mice with Coxsackie B3 virus-induced myocarditis [171]. APEX1 levels were found to increase progressively, maintain for an extended period and correlate positively with myocardial inflammation, thus reflecting the severity of myocardial injury [172].

APEX1 also has a considerable role in myocardial infarction. Polymorphism of human APEX1 exon 5 148 Asp(A)/Glu(G) has been reported in patients with myocardial infarction. 47% (21 out of 45) of patients undergoing first-time elective coronary artery bypass grafting carry an APEX1 148 A/G variant, which is observed in only 18% (7 out of 40) healthy controls [173]. Interestingly, the APEX1 148 A-to-G substitution is associated with irradiation-induced G2 cell cycle arrest in human lymphocytes [174], suggesting this variant has an impaired DNA repair capacity. Although the association of APEX1 exon 5 polymorphisms with myocardial infarction requires validation with a much larger cohort, this line of human genetic evidence suggests a role of APEX1 BER activity in the susceptibility to myocardial infarction. Supportively, the transplant of Sca1+ cardiac progenitor cells overexpressing APEX1 into infarcted mouse hearts improves the survival of transplanted cells and host cardiomyocytes. This transplant improves left ventricle ejection fraction, correlated with increased border zone angiogenesis, suppressed cardiac fibrosis, reduced infiltration of M1-polarized macrophages, and reduced myocardial cytokine (IL-1β, IL-6) expression. An in vitro co-culture of primary mouse cardiac progenitor cells overexpressing APEX1 reduces the anoxia-induced apoptosis of neonatal rat ventricular myocytes. In addition, the conditioned medium of APEX1-expressing cardiac progenitor cells enhanced the angiogenic behaviors of HUVECs, partially attributable to the increase in paracrine VEGF-A. These findings highlight an important, potentially therapeutic role of APEX1 in the intercellular coordination of cardiac repair following ischemic injury [175].

## 5. Conclusions

Collectively, emerging evidence underscores APEX1 as a crucial regulator of cardiovascular function through its roles in DNA repair and redox signaling. Its contributions to counteracting oxidative stress, limiting neointimal lesion formation, and influencing aging processes highlight a potentially valuable therapeutic avenue. While studies suggest that upregulating APEX1 can lower pro-inflammatory cytokine levels and bolster protective mechanisms in cardiovascular tissues, a more comprehensive understanding of its precise roles—in various cell types and at distinct disease stages—remains a priority for future research.

Despite these promising observations, important gaps persist. DNA damage has been implicated in diverse cardiovascular pathologies, including congenital heart disease, diabetes-related heart dysfunction, atherosclerosis driven by mechanical stress, and heart failure. However, the specific involvement of APEX1 in these contexts, especially for congenital and diabetic heart disease or endothelial dysfunction under disturbed flow, has yet to be fully elucidated. Given APEX1’s recognized capacity to maintain genomic stability through its base excision repair function, targeted modulation of this enzyme might pave the way for novel treatments in cardiovascular medicine.

Moving forward, further in-depth clinical and experimental studies are essential. Dissecting how APEX1 mediates cytoprotection and antiinflammatory effects across different cardiovascular disease models will clarify whether enhancing its expression or activity could serve as an effective intervention. Such investigations will help to integrate APEX1 more firmly into our therapeutic armamentarium for combatting cardiovascular diseases.

## Data Availability

No new data were created or analyzed in this study. Data sharing is not applicable to this article.

## List of Abbreviations

AIM	absent in melanoma
AIM2	absent in melanoma 2
Ang II	angiotensin II
AP	apyrimidinic
AP-1	activator protein 1
APEX1	apurinic/apyrimidinarric endonuclease 1
AP-site	apyrimidinic site
AS	atherosclerosis
BER	base excision repair
BMPR2	bone morphogenetic protein receptor type 2
BPAEC	bovine pulmonary arterial endothelial cells
CASP1	caspase 1
CVD	cardiovascular diseases
Cytc	cytochrome c
DNA-pol	DNA polymerase
DSR-Pathway	double-strand break repair pathway
EC	endothelial cell
eNOS	endothelial NO synthase
ER	endoplasmic reticulum
ERK1/2	extracellular regulated protein kinases 1/2
ESC	embryonic stem cell
Exo III	endonuclease III
GAPDH	glyceraldehyde 3-phosphate dehydrogenase
GDNF	glial cell derived neurotrophic factor
GLP-1	glucagon-like peptide-1
GSEA	gene enrichment analysis
GSMDM	gasdermin
GSMDM N-terminal	N-terminal of GSDMD
H/R	hypoxia/reoxygenation
HDAC	histone deacetylase
HEK 293	human embryonic kidney cells
HF	Heart Failure
HULEC-5a	human lung microvascular endothelium cells
HUVECs	human umbilical vein endothelial cells
IL-1β	interleukin-1 beta
IL-6	interleukin-6
iNOS	inducible nitric oxide synthase
Htra3	htra serine peptidase 3
KO	knockout
LARP7	la ribonucleoprotein 7
LCBⅠ	microtubule associated protein 1 light chain 3 alpha
LCBⅡ	microtubule associated protein 1 light chain 3 beta
LDH	lactate dehydrogenase
LPS	lipopolysaccharide
mt-DNA	mitochondrial DNA
NADPH	nicotinamide-adenine dinucleotide phosphate
NEDD4	neuronally expressed developmentally downregulated 4
NF-κB	nuclear factor kappa-B
NLS	nuclear localization signal
NO	nitric oxide
NPM1	nucleophosmin1
oxLDL	oxidized low-density lipoprotein
P21	cyclin dependent kinase inhibitor 1A
P53	tumor protein P53
PARP-1	poly (ADP-ribose) polymerase 1
PIT1	pituitary-specific positive transcription factor
PINK1	PTEN induced putative kinase 1
PKC	protein kinase C
PMA	propidium monoazidos
Pol	polymerase
PPAR-γ	peroxisome proliferator activated receptor γ
PPI	protein-protein interaction
PTM	protein translational modification
ROS	reactive oxygen species
ROS	reactive oxygen species
RUNX2	runt-related transcription factor 2
SIRT1	silent mating type information regulation 2
SNP	single nucleotide polymorphism
ss-DNA	single-stranded DNA
SSR-Pathway	single-strand break repair pathway
STAT3	signal transducer and activator of transcription 3
TGFβ	transforming growth factor beta
TNF	tumor necrosis factor
VCAM	vascular cell adhesion molecule
VCAM-1	vascular cell adhesion molecule 1
VEGF	vascular endothelial growth factor
VSMC	vascular smooth muscle cell
WT	wide type
XRCC1	X-ray repair cross-complementing 1
17-DMAG	alvespimycin
17-AGG	tanespimycin
AUY-922	luminespib
